# Predictive Blood Chemistry Parameters for Pansteatitis-Affected Mozambique Tilapia (*Oreochromis mossambicus*)

**DOI:** 10.1371/journal.pone.0153874

**Published:** 2016-04-26

**Authors:** John A. Bowden, Theresa M. Cantu, Robert W. Chapman, Stephen E. Somerville, Matthew P. Guillette, Hannes Botha, Andre Hoffman, Wilmien J. Luus-Powell, Willem J. Smit, Jeffrey Lebepe, Jan Myburgh, Danny Govender, Jonathan Tucker, Ashley S. P. Boggs, Louis J. Guillette

**Affiliations:** 1 National Institute of Standards and Technology (NIST), Material Measurement Laboratory, Chemical Sciences Division, Environmental Chemical Sciences Group, Hollings Marine Laboratory, Charleston, South Carolina, United States of America; 2 Departments of Obstetrics and Gynecology, Medical University of South Carolina (MUSC), Charleston, South Carolina, United States of America; 3 Marine Resources Research Institute, South Carolina Department of Natural Resources, Hollings Marine Laboratory, Charleston, South Carolina, United States of America; 4 Scientific Services, Mpumalanga Tourism and Parks Agency, Nelspruit, South Africa; 5 Department of Biodiversity, University of Limpopo, Sovenga, South Africa; 6 Department of Paraclinical Sciences, Faculty of Veterinary Science, University of Pretoria, Onderstepoort, South Africa; 7 Scientific Services, South African National Parks, Skukuza, South Africa; James Cook University, AUSTRALIA

## Abstract

One of the largest river systems in South Africa, the Olifants River, has experienced significant changes in water quality due to anthropogenic activities. Since 2005, there have been various “outbreaks” of the inflammatory disease pansteatitis in several vertebrate species. Large-scale pansteatitis-related mortality events have decimated the crocodile population at Lake Loskop and decreased the population at Kruger National Park. Most pansteatitis-related diagnoses within the region are conducted post-mortem by either gross pathology or histology. The application of a non-lethal approach to assess the prevalence and pervasiveness of pansteatitis in the Olifants River region would be of great importance for the development of a management plan for this disease. In this study, several plasma-based biomarkers accurately classified pansteatitis in Mozambique tilapia (*Oreochromis mossambicus*) collected from Lake Loskop using a commercially available benchtop blood chemistry analyzer combined with data interpretation via artificial neural network analysis. According to the model, four blood chemistry parameters (calcium, sodium, total protein and albumin), in combination with total length, diagnose pansteatitis to a predictive accuracy of 92 percent. In addition, several morphometric traits (total length, age, weight) were also associated with pansteatitis. On-going research will focus on further evaluating the use of blood chemistry to classify pansteatitis across different species, trophic levels, and within different sites along the Olifants River.

## Introduction

The Olifants River system, within the Mpumalanga Province in South Africa, is known to be highly contaminated by various anthropogenic sources, mostly from the upper and middle catchments, as a result of agricultural, mining, manufacturing, and water treatment practices [1–3]. Lake Loskop collects drainage from approximately 11,464 km^2^ of land area in the upper catchment of the Olifants River system and, within the past 30 years, has experienced a severe decline in its Nile crocodile (*Crocodylus niloticus*) population, along with several instances of dramatic fish mortality [4–8]. In 1979, crocodile surveys at Lake Loskop indicated an estimated 32 animals; however, this number has dropped dramatically to as few as 4 animals in 2010 [3, 4]. The current population also appears to include only smaller crocodiles (below reproductive age), as opposed to an equal distribution among different size classes [3, 9]. Other portions of the Olifants River system have similarly witnessed massive mortality events of crocodiles and fish. In 2008, approximately 180 adult crocodile carcasses were discovered in the Olifants River Gorge in Kruger National Park (KNP), some 530 km downstream from Lake Loskop, with an estimated number of 500 individual deaths in total [2, 6, 7, 10–13]. The next season (2009), approximately 24 crocodile carcasses were observed in the same area [14]. Hypothesized causes for these significant mortality events include direct impacts from increased water pollution from local mines and agriculture, algal blooms, and alteration/destruction of habitat in areas impacted by dams [1, 15–17]. Beyond isolating and connecting specific point source(s) to the large-scale mortality events in the Olifants River system (2003–2010), veterinary examination concluded that the ultimate cause of death in these events was an environmental form of pansteatitis, a disease noted by the presence of inflammation in adipose tissue [11, 14]. In addition to crocodile deaths, other species have presented pansteatitis and/or suffered mortality events, including the African sharptooth catfish (*Clarias gariepinus*), Rednose labeo (*Labeo rosae*), Mozambique tilapia (*Oreochromis mossambicus*) and serrated hinged terrapins (*Pelusios sinuatus*) [2, 6, 10, 13, 18]. Fish-eating waterfowl species in the Olifants River system have been affected as well; African fish eagles (*Haliaeetus vocifer*) in the Lake Loskop area have struggled to reproduce and cormorant numbers are on the decline [5]. In KNP, there has been a 35% reduction in the African fish eagle population since 1992, and herons have become rare on the Olifants River [10]. The widespread geographical, species-specific, and temporal range of pansteatitis described in the Olifants River system elicits concern in respect to its overall impact on tourism, ecology, and both human and environmental health in the region.

While diets high in unsaturated fatty acids and/or low in vitamin E have been identified as causative agents of pansteatitis in domestic or farmed animals [19–22], several hypotheses for the triggers of pansteatitis in the environment have included exposure to anthropogenic contaminants, introduction of invasive species, exposure to cyanotoxins, consumption of improper diet, and changes in water chemistry and/or parasites; however, none have been identified as the definitive cause of pansteatitis in either crocodiles, fish, or birds [1, 8, 13–16, 20, 23–25]. It is interesting to note that these suggested causes could all be interconnected, as the introduction of approximately 200 water storage dams to the Olifants River system has changed the ecology of the river, possibly leading to a cascade of effects up the trophic pyramid [3]. While large mortality events appear to have tapered off in recent years, it is currently unknown whether pansteatitis is pandemic in the Olifants River region. Current work identifying the disease with varying consistency in multiple species across different trophic levels without a definitive trigger of disease suggests a critical need for increased efforts to examine the overall presence of pansteatitis in the Olifants River system. Currently, there are no reports available that highlight the prevalence and severity of pansteatitis in fish species within the Olifants River system, which is an integral component needed to begin the process of characterizing the disease within the region. Establishment of expansive population-based diagnostic surveys across potentially affected species, sites, and age classes would be a critical advancement to providing the first assessment of impact of pansteatitis within the region.

One reason for the lack of routine disease diagnostic surveys is the considerable amount of effort, financial and logistical, required to adequately examine affected species in the entire Olifants River system. The catchment area covers an approximate area of 74,500 km^2^, with many of the regions too remote to set up mobile laboratories. Another reason for the paucity of routine disease diagnostic surveys is that current diagnosis of pansteatitis remains contingent upon examination during necropsy, which is problematic for population-wide assessment of living animals. Blood chemistry analysis has proven to be an effective approach in providing a non-lethal population-based assessment of environmental health and disease [26–32]. Investigating the current health status of sentinel species within pansteatitis-affected regions in the Olifants River system via blood chemistry would represent an integral first step in providing population-based disease assessments. Further, selection of a relevant sentinel species (e.g. fish) to examine potential blood chemistry markers should be considered. Fish exhibited parallel mortality events to the crocodile, are easier to catch, certain species (Mozambique tilapia) are not classified as either endangered or threatened, and therefore present several benefits as a relevant sentinel species in the assessment of newly developed diagnostic assays for pansteatitis. Tilapia were chosen at Lake Loskop due to their relatively robust population and high incidence of pansteatitis [2, 8].

Blood chemistry, using benchtop analyzers, has been used previously to define baseline levels to assess population health in the environment [33–41] and to assess potential contaminant exposure [42–46] or prevalence of disease [47–50] in both laboratory and environmental-based settings. In this study, Mozambique tilapia were sampled from Lake Loskop, Mpumalanga, South Africa, one of the ground zero sites of pansteatitis and a site of significant population decline for the Nile crocodile [4], in an effort to evaluate the use of plasma as a suitable matrix for discovery of pansteatitis-related biomarkers. Specifically, blood chemistry levels for aspartate aminotransferase (AST), bile acids (BA), creatine kinase (CK), uric acid (UA), glucose (GLU), inorganic phosphorous (PHOS), calcium (Ca^2+^), total protein (TP), albumin (ALB), globulin (GLOB), potassium (K^+^), and sodium (Na^+^) in tilapia samples from Lake Loskop were examined using a commercially available benchtop blood chemistry analyzer. In this study, significant differences were examined between healthy and pansteatitis-affected tilapia for each blood chemistry parameter and morphometric trait (e.g., age, total length, weight). These findings were further scrutinized using artificial neural network (ANN) analyses, in an effort to develop and validate a statistical model that could accurately classify disease status using the blood chemistry results. To accompany the data, a discussion is provided examining the merits of using a blood chemistry analyzer as an environmental disease state diagnostic tool.

## Materials and Methods

### Fish Collection

The project proposal (ES 6/1) was peer reviewed by MTPA Scientists. The peer review found the project concept, motivation and aims to be well defined and scientifically acceptable and found the proposed methods to be within acceptable ethical standards. The peer reviewers recommended that the project and its ethics be approved and implemented. The project met the permitting and ethical guidelines (which include adherence to legal requirements) of the study country. The studies did not involve endangered or protected species. All fish work was conducted using the permit and animal handling protocol that was reviewed and approved by the Mpumalanga Tourism and Parks Agency (Project #ES 6/1).

Mozambique tilapia (n = 39) were collected from Lake Loskop (S 25.453161, E 29.294759), Mpumalanga, South Africa, between July 28 and August 1, 2014 (pre-spawning period). Water chemistry parameters at the site of fish collection were recorded using a PCSTestr 35 (Oakton Instruments, Vernon Hills, IL). Multiple measurements were obtained during sample collection and the parameter values were: water temperature (15.6 ± 1.2°C), pH (9.07 ± 0.48), conductivity (350 ± 3 μS), total dissolved solids (248 ± 2 ppm), and salinity (166 ± 1 ppt). Fish (≈ 40 cm) were randomly caught using 8-inch mesh gill nets (on average 25 min in net prior to removal). A standardized gill netting approach was employed due to its ability to efficiently provide a high number of quality samples. All capture methods incur some level of stress for the animal (which have the potential to influence blood chemistry), thus it should be noted that the resultant blood chemistry values may reflect both capture stress and health/disease status. However, even if the blood chemistry responses that are observed are related to the difference of how pansteatitis-affected and healthy animals handle stress, this diagnostic approach would still be a useful tool, as the ultimate goal of this study was to find a way to non-lethally classify disease status.

**Fig 1:**
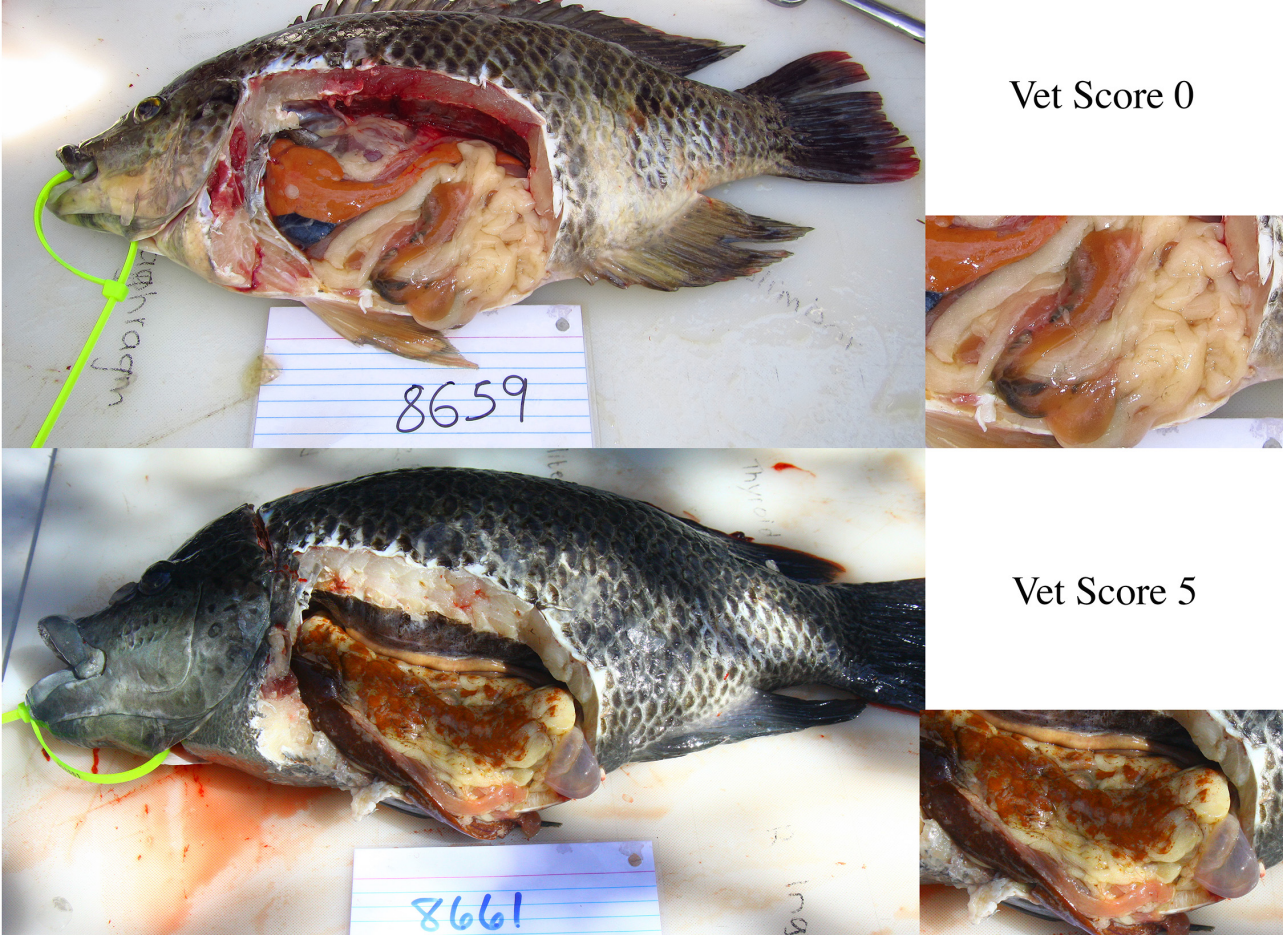
Visual examination of healthy (top) and pansteatitis-affected tilapia (bottom), enhanced images on the right. The examples shown are male tilapia. Note the lesions on the diseased tilapia (orange lesions, bottom right photo).

Fish were tagged with a unique identification marker (through the dorsal fin), and an immediate blood sample was obtained via caudal venipuncture from the lateral line. Whole blood was collected from the lateral line in 10 mL lithium heparin tubes (BD Medical, Franklin Lakes, NJ) and stored on ice until centrifugation. Plasma was obtained by centrifugation (10 min at 120 rad/s) using a tabletop centrifuge, distributed into pre-labeled 2 mL cryovials, and flash frozen in liquid nitrogen until analyzed. There was no indication of hemolysis or lipemia observed during blood draw. Immediately after blood sampling, prior to euthanasia, the animals were transported from boat to mobile laboratory in cold water tanks with oxygen [51]. Fish were decapitated with a sharpened filet knife immediately followed by pithing of the brain, or destruction of the brain tissue. During necropsy (n = 19 males, n = 20 females), fish were sexed and assigned health scores on a 0–5 scale (zero indicating no disease) by at least two veterinarians on-site (individual scores documented were the average of multiple independent veterinarians’ scores and the final scores are shown in S1 Table). Scores were determined using criteria centered on the number, size, and color of the lesions present in the adipose tissue. A visual example of both a healthy (0 score) and pansteatitis-affected tilapia (5 score) is shown in Fig 1. For the purpose of evaluating blood chemistry values as potential biomarkers of pansteatitis, the most appropriate designation for healthy and pansteatitis-affected tilapia was with a score of < 1 and with a score ≥ 1, respectively. It should be noted that those tilapia with a score of 0 had absolutely no presentation of the disease, while those with a score of 0.5 had extremely small/subtle lesions with a majority of the adipose unaffected (also classified as healthy by the veterinarians on-site). In total, there were 9 and 9 healthy male and female tilapia, respectively, and 10 and 11 pansteatitis-affected male and female tilapia, respectively.

Additional information (S1 Table) obtained during fish sampling included sex of the fish, body mass (hanging scale, kg), and total length (ruler board, cm). Otoliths were collected from each fish to determine age.

### Blood Chemistry Analysis

Blood chemistry values were obtained using an Abaxis VetScan Whole Blood chemistry analyzer with Avian/Reptilian Profile Plus rotors (as recommended by Abaxis, 500–0041, Abaxis, Union City, CA). The avian/reptile rotor has been previously used for measurement of blood chemistry parameters in various fish species [38, 39]. The instrument was used in accordance with the procedures noted in the Abaxis VetScan analyzer manual. In brief, 150 μL of plasma was volumetrically loaded onto a disposable avian/reptile rotor and analyzed. The blood chemistry parameters examined using the avian/reptilian rotors included (with instrument range, [52]): AST (5 to 2000 U/L or 83.35 to 33340 nano katal/L), TP (2 to 14 g/dL), ALB (1 to 6.5 g/dL), GLOB (0 to 13 g/dL), GLU (10 to 700 mg/dL), PHOS (0.2 to 20 mg/dL), K^+^ (1.5 to 8.5 mmol/L), Na^+^ (110 to 170 mmol/L), Ca^2+^ (4 to 16 mg/dL), BA (35 to 200 μmol/L), CK (5 to 14000 U/L or 83.35 to 233380 nano katal/L), and UA (0.3 to 25 mg/dL). Total analysis time for each sample was approximately 15 min. The raw blood chemistry values for each blood chemistry parameter detected in each tilapia plasma sample (n = 39) is shown in S2 Table (male) and S3 Table (females).

### Quality Control

To determine the practicality of using avian/reptile rotors for examining fish blood chemistry parameters, a species-matched (tilapia) QC sample was investigated with multiple replicates (n = 7) as described above. The species-matched QC sample was constructed from 12 randomly selected tilapia caught during a concurrent active sampling experiment (using same handling procedures as previously described). Using the avian/reptile rotor, the blood chemistry analyzer was able to provide values for 11 of the 12 parameters using the species-matched QC sample, as shown in S4 Table. Uric acid was not detected in the tilapia QC sample. The reproducibility (relative standard deviation, RSD) of this trial (n = 7) was at or below 3% for all parameters detected, except for BA (9%), CK (6%), and K+ (4%).

To assess the precision and accuracy of the blood chemistry parameter measurements during sample analysis, Standard Reference Material (SRM) 1950 Metabolites in Frozen Human Plasma, was examined (n = 7) randomly and the determined values were compared to the noted values on the Certificate of Analysis (COA) [53]. Despite being human plasma, the blood chemistry analyzer was able to provide values on 11 of the 12 parameters (with BA the only parameter which did not yield a value, as shown in S5 Table). The reproducibility of the SRM measurements was at or below 4% for all parameters detected, except for AST (7%), CK (7%), and PHOS (7%).

### Fish Age via Otolith Examination

Tilapia have thin sagittal otoliths in the medial-distal plane, and thus, transverse sectioning was chosen for age determination. A pencil line was drawn across the concave side (distal) of the otolith through the core to mark the transverse section. The otolith was then embedded in an epoxy resin in a silicone mold and was allowed to set overnight. After the epoxy had set and the otolith was removed from the mold, the embedded otolith was glued to a paper tab with the pencil mark orientated to align with the cutting blades. When positioned into an Isomet saw chuck, with two diamond wafer blades (with a 0.4 mm spacer used to cut the section of otolith), the otolith was cut where the pencil mark lined up between the blades. The resultant section was carefully removed and cleaned with a 70% isopropyl solution (volume fraction, UNIVAR, Redmond, WA). The cleaned otolith section was then secured to a microscope slide with Cytoseal XYL (Richard-Allan Scientific, Kalamazoo, MI) for improved readability and the annuli were counted using a dissecting microscope. Two independent readers confirmed the age determination of each fish.

### Statistics and Artificial Neural Network

Initial statistical examination of the blood chemistry data (and morphometric traits) focused on employing two-tailed t-tests to assess significance between healthy (vet score < 1) and pansteatitis-affected tilapia (vet score ≥ 1) using JMP software (v.11.2.1, SAS, Cary, NC). Prior to determining significance (t-tests, p < 0.05), the blood chemistry data (and physical attributes) were tested for normality using a Shapiro-Wilk test and equal variances using a Levene test. If data did not fit these assumptions, Mann-Whitney U tests were employed (χ^2^ < 0.05). When comparing healthy and pansteatitis-affected animals, it was also important to determine if sexual dimorphism was present in the blood chemistry data (and morphometric traits). If sexual dimorphism was not observed, sexes were combined to increase power of analysis; however, if sexual dimorphism was observed, then the significance was determined for each sex independently.

To model the combinatorial effects of the blood chemistry panel (12 measured parameters) using the blood chemistry analyzer and to evaluate its strength as a disease diagnostic approach for pansteatitis, a supervised machine learning algorithm, or more specifically, a feed forward artificial neural network (ANN), was employed using a customized MATLAB program (v.R2014b). It is important to note that both sexes were combined to construct the ANN model in an effort to increase sample size. To begin, 20 ANN models were constructed from the tilapia blood chemistry data (with age and total length included) by randomly sampling 70% of the records and extracting the model fit (R-square) and sensitivity of the disease status prediction to changes in each of the diagnostic variables. This process was analogous to extracting the coefficient of variation in linear regression. The disease status of the remaining samples (30%) were then predicted, which were not used in the ANN training session, as a cross validation set (CV) to test the robustness of the ANN training by extracting the R-squares for this data set. The next step was to construct receiver operating characteristic (ROC) curves by using the observed and predicted disease statuses of the CV data. Following this initial testing of the data, four of the blood chemistry variables with the strongest impact (highest sensitivities) on correct diagnoses were selected (Ca^2+^, TP, ALB, and Na^+^) and the analysis was repeated using only these four input variables with total length (age was removed due to lethality of otolith collection). Surface plots for the top four predictive parameters (Ca^2+^, Na^+^, ALB, and TP) in relation to vet score and total length (while the other three parameters were clamped) were created. Surface plots were also created examining the four predictive parameters (Ca^2+^, Na^+^, ALB, and TP) in relation to standard deviation and total length (while the other three parameters were clamped) to assess weaknesses in our sample set distribution. It should be noted that sample 8683 was not used in the ANN analysis due to the lack of a calculated CK value obtained using the blood chemistry analyzer.

## Results

For quality control purposes and to assess the accuracy of the blood chemistry analyzer using the avian/reptile rotor, SRM 1950 was added to the study to further examine the measurement accuracy of each blood chemistry parameter, as 10 of the 11 parameters detected using the blood chemistry analyzer (excluding PHOS) had either certified, reference, or online values for comparison purposes [53, 54]. The average measured values (n = 7) using the blood chemistry analyzer for ALB, K^+^ and GLU in SRM 1950 were measured within the certified confidence interval, as shown in S6 Table. Total protein, which is listed as a reference value, was also within the confidence interval. Two parameters (Na^+^ and UA), for which certified values are available, were measured below the confidence interval. For Na^+^, the measured value was only slightly below the certified value (-3% difference), while UA was well below the certified value (-22% difference). For UA, the high percent difference between the measured and certified value could be an artifact of measuring mammalian plasma on an avian/reptile specific rotor. Nevertheless, since UA was not detected with the tilapia samples, this aspect was not investigated further. Three parameters (AST, CK and ALB) had comparison values (provided by contributing laboratories) on the SRM/D website and the average values listed on the site for SRM 1950 were compared to the average values measured in this study. Using percent difference, the measured values were all approximately 10% higher or less than the noted SRM/D values. Lastly, the value for GLOB, which is calculated by the blood chemistry analyzer by subtracting ALB from the TP value was calculated similarly using the noted SRM 1950 values. The resultant calculated value for GLOB for SRM 1950 was 2.31 g/L. The blood chemistry analyzer reported a GLOB value of 2.10 g/L, which resulted in a percent difference of approximately 9.5% (shown in S6 Table).

The mean ± standard error of the mean (SEM) for each blood chemistry parameter for both healthy and pansteatitis-affected tilapia for each sex (M/F) and both sexes combined are shown in Table 1. BA was excluded from further interpretation as several tilapia samples did not register a measured value. Using t-tests or Mann-Whitney U tests when appropriate and the previously described health score designation (healthy < 1, pansteatitis-affected ≥ 1), each blood chemistry parameter was investigated for statistical significance between healthy and pansteatitis-affected tilapia groups (with significance having p or χ^2^ < 0.05), as shown in Table 2. For the parameters that were determined to not be sexually dimorphic (AST, CK, GLU, TP, GLOB, K^+^ and Na^+^), only TP (χ^2^ < 0.0001), GLOB (p < 0.0001), and Na^+^ (p = 0.0116) were significantly different when comparing healthy and pansteatitis-affected tilapia. The results (combined sexes) indicate that the healthy animals have higher measured values in comparison to the pansteatitis-affected counterparts.

**Table 1:** Mean and SEM values for healthy and diseased tilapia obtained using the blood chemistry analyzer.

	*Male Healthy (n = 9)*	*Male Diseased (n = 10)*	*Female Healthy (n = 9)*	*Female Diseased (n = 11)*	*Healthy (n = 18)*	*Diseased (n = 21)*
AST	75 ± 22	64 ± 18	115 ± 47	61 ± 15	95 ± 26	62 ± 11
CK	1258 ± 500	2123 ± 450	2203 ± 680	2079 ± 420	1832 ± 440*	2100 ± 300
GLU	43 ± 7	32 ± 2	34 ± 2	36 ± 4	38 ± 4	34 ± 2
Ca^2+^	14.5 ± 0.2	12.2 ± 0.3	15.9 ± 0.6	14.8 ± 0.7	15.2 ± 0.3	13.6 ± 0.5
PHOS	8.0 ± 0.4	5.1 ± 0.3	7.6 ± 0.5	6.4 ± 0.4	7.8 ± 0.3	5.8 ± 0.3
TP	3.9 ± 0.1	3.1 ± 0.1	4.0 ± 0.2	3.3 ± 0.1	3.9 ± 0.1	3.2 ± 0.1
ALB	2.1 ± 0.1	1.6 ± 0.1	2.2 ± 0.1	2.0 ± 0.1	2.2 ± 0.1	1.8 ± 0.1
GLOB	1.8 ± 0.1	1.5 ± 0.1	1.8 ± 0.1	1.3 ± 0.1	1.8 ± 0.1	1.4 ± 0.0
K^+^	4.3 ± 0.2	4.1 ± 0.2	4.4 ± 0.3	4.1 ± 0.1	4.3 ± 0.2	4.1 ± 0.1
Na^+^	172 ± 2	162 ± 3	170 ± 2	166 ± 3	171 ± 1	164 ± 2

Values provided are the mean ± SEM.

* Indicates that the CK value for sample 8683 was not included in mean due to the lack of a calculated CK value obtained using the blood chemistry analyzer.

**Table 2:** Examination of blood chemistry parameters and morphometric traits using t-tests or Mann-Whitney U tests.

	Healthy M vs. Diseased M	Healthy F vs. Diseased F	Healthy vs. Diseased (Combined Sex)	Comments
	*n = 9 vs*. *n = 10*	*n = 9 vs*. *n = 11*	*n = 18 vs*. *n = 21*	
AST			χ^2^ = 0.1390	No significant difference
CK			χ^2^ = 0.3706	No significant difference
GLU			χ^2^ = 0.3517	No significant difference
Ca^2+^	**χ**^**2**^ **= 0.0004**	χ^2^ = 0.1016		Healthy M had higher Ca^2+^
PHOS	**p < 0.0001**	p = 0.0815		Healthy M had higher PHOS
TP			**χ**^**2**^ **< 0.0001**	Healthy had higher TP
ALB	**p < 0.0001**	**p = 0.0091**		Healthy had higher ALB
GLOB			**p < 0.0001**	Healthy had higher GLOB
K^+^			χ^2^ = 0.2912	No significant difference
Na^+^			**p = 0.0116**	Healthy had higher Na^+^
Age	**p = 0.0025**	**p < 0.0001**		Diseased were older
Total Length	**χ**^**2**^ **= 0.0033**	**χ**^**2**^ **= 0.0086**		Diseased were longer
Weight	**χ**^**2**^ **= 0.0142**	χ^2^ = 0.0608		Diseased M were heavier

Significance was determined (in bold) by calculated p- and χ^2^-values shown (significance < 0.05). Abbreviations for each blood chemistry parameters: aspartate aminotransferase (AST), bile acids (BA), creatine kinase (CK), uric acid (UA), glucose (GLU), inorganic phosphorous (PHOS), calcium (Ca^2+^), total protein (TP), albumin (ALB), globulin (GLOB), potassium (K^+^), and sodium (Na^+^). BA and UA were not examined.

Sexual dimorphism of the blood chemistry parameters (and morphometric traits) was examined by evaluating significance between males and females using the healthy and pansteatitis-affected classification. The three blood chemistry parameters that exhibited sexual dimorphism (healthy: pansteatitis-affected) were Ca^2+^ (p = 0.0415: p = 0.0030), ALB (p = 0.2719: p = 0.0019) and PHOS (p = 0.5618: p = 0.0158). In all cases with a calculated significance between male and female groups, females had higher measured values in comparison to the male counterpart. Thus, statistical significance between healthy and pansteatitis-affected tilapia for these three blood chemistry parameters was performed for each sex independently, as shown in Table 2. For males, it was found that Ca^2+^ (χ^2^ = 0.0004), PHOS (p < 0.0001), and ALB (p < 0.0001) were all significantly different when comparing healthy and pansteatitis-affected individuals. For females, only ALB (p = 0.0091) was significantly different between healthy and pansteatitis-affected individuals. In all cases where significance was observed, the healthy animals had higher measured values in comparison to the pansteatitis-affected individuals. Based on these results, different ranges of blood chemistry parameters (Ca^2+^, ALB, and PHOS) for healthy and pansteatitis-affected should be expected between male and female tilapia.

The morphometric traits of both the healthy and pansteatitis-affected tilapia were also explored, and included age, total length and weight, which has been previously shown to exist [55]. As with the blood chemistry analysis, sexual dimorphism was investigated first for each morphometric trait. Sexual dimorphism was observed for all measured traits. Significance between males and females using the healthy and pansteatitis-affected classification (healthy: pansteatitis-affected) was determined for age (males p = 0.0301: females p = 0.0081), total length (χ^2^ = 0.1212: χ^2^ = 0.0012) and weight (p = 0.1856: χ^2^ = 0.0005). In summary, females were older and males were longer and heavier (within the population examined in this study). Thus, due to the sexual dimorphic nature of these traits, statistical significance between healthy and pansteatitis-affected tilapia was performed for each sex independently, as shown in Table 2. Surprisingly, a significant difference (p or χ^2^ < 0.05) between healthy and pansteatitis-affected tilapia was found for each morphometric trait. A significant difference was observed (male p = 0.0025, female p < 0.0001) for the age (derived via otolith examination) of both healthy and pansteatitis-affected tilapia, as shown in Table 2. The mean age (year) was 5.5 and 6.4 for healthy males and females, respectively, while the mean age (year) was 7.5 and 9.6 for pansteatitis-affected males and females, respectively. On average, pansteatitis-affected male and female tilapia were 30% and 40% older, respectively, when compared to healthy counterparts. A significant difference was observed (male χ^2^ = 0.0033, female χ^2^ = 0.0086) when investigating the total length of both healthy and pansteatitis-affected tilapia. Total length (cm) followed a similar trend to age; as typically the longer fish (on average 42.4 cm) were statistically more diseased than tilapia with smaller total length (on average 39.4 cm), as shown in Table 2. More specifically, when comparing the total length between healthy and pansteatitis-affected tilapia, diseased male and female tilapia were approximately (on average) 10% and 6% longer, respectively, compared to the healthy tilapia. A significant difference was only observed in males (χ^2^ = 0.0142) for total weight (kg). The mean weights for healthy males and females were 1.5 and 1.3, respectively, while mean weights for pansteatitis-affected males and females were 2.0 and 1.5, respectively.

**Fig 2:**
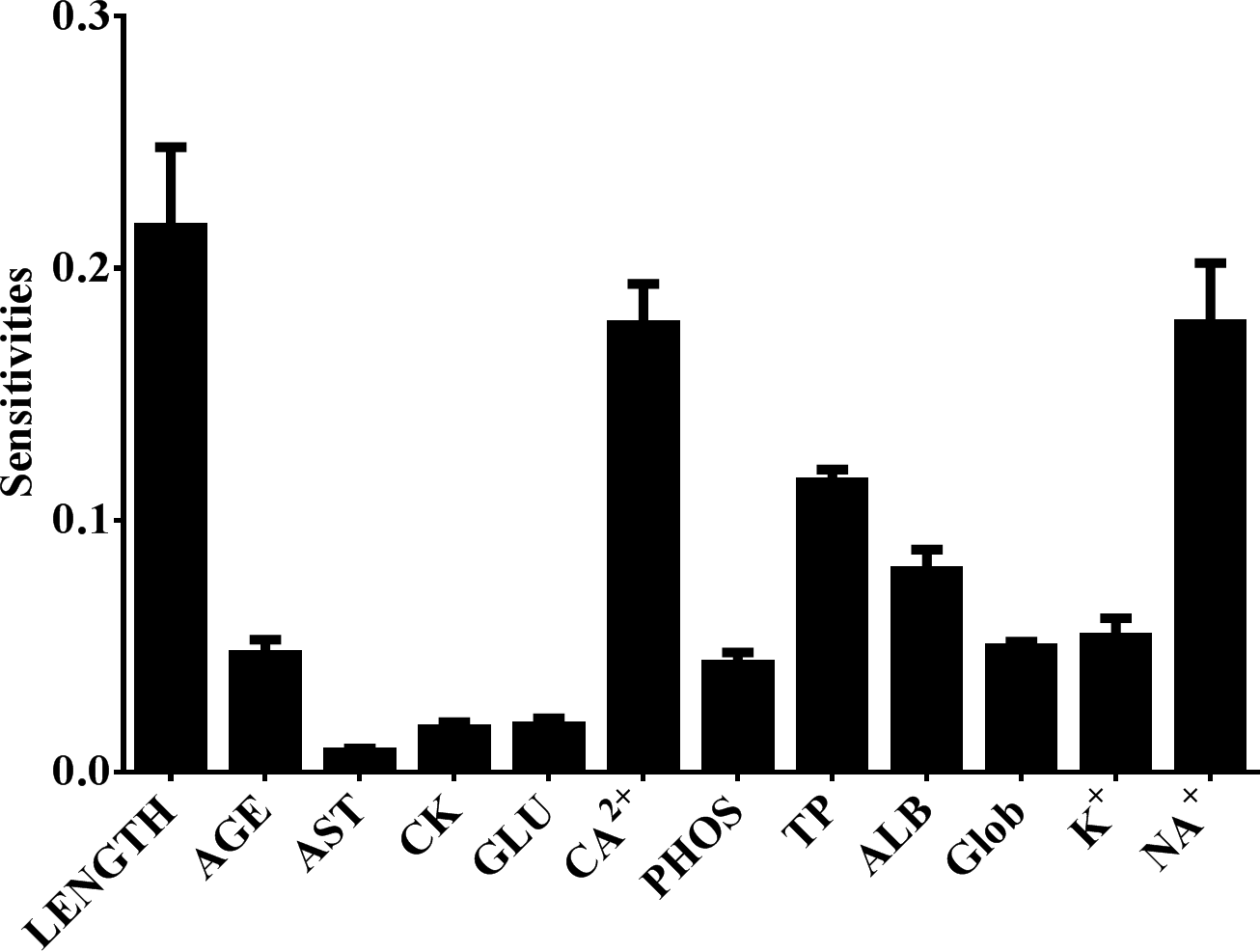
Sensitivities plot for assessing which variables were most important for classifying pansteatitis. Error values are shown as standard deviation of the mean.

An ANN model was employed to evaluate the predictive nature of the blood chemistry profile between healthy and pansteatitis-affected tilapia, which used a subset of the samples (70%, comprised of both sexes) to construct a disease-classifying model. The model was constructed utilizing both the physical characteristics (age, total length) of each fish and the blood chemistry data. The R-squared value for the ANN model, or the fit of the data used to train the ANN, was 0.93 ± 0.03. To test the efficacy of this model in predicting pansteatitis, the remaining sample data (30%) was tested using the constructed model. The R-squared CV value was 0.45 ± 0.08, suggesting that the model was fairly robust in explaining variation within the data set. To assess which blood chemistry parameters were most important to correctly classify pansteatitis, a sensitivities plot was generated (Fig 2). As shown in Fig 2, the principle drivers for correct classification of pansteatitis were total length, TP, ALB, Ca^2+^ and Na^+^. Total protein, ALB, and Na^+^ were expected (from t-test results); however, Ca^2+^ was not expected as no statistical significance was shown for females using t-tests (Table 2). Sex, which is not included in the sensitivity plot shown in Fig 2, was examined in previous models and was shown to be a very small factor in classifying disease (sensitivity value was lower than all blood chemistry parameters, except CK, data not shown).

**Fig 3:**
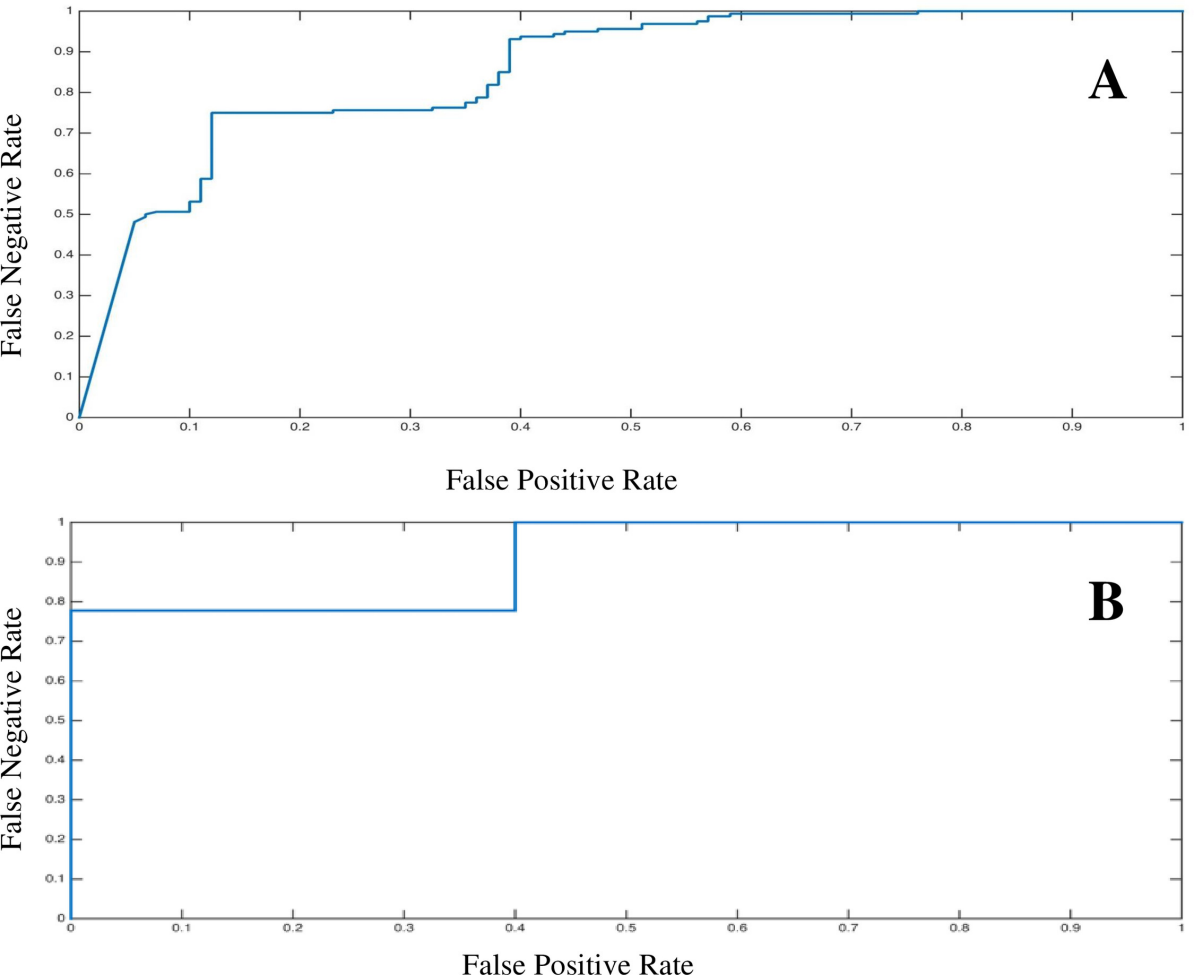
**(A) ROC curve using all the blood chemistry parameters with age and total length included.** The area under the curve was 0.85 with a standard error of 0.02. **(B) ROC curve using the four most important blood parameters: Ca**^**2+**^**, Na**^**+**^**, TP and ALB with total length.** The area under the curve was 0.92 with a standard error of 0.06.

ROC curves were generated (with area under the curve representing the calculated predictive capability of the ANN model) to assess the ability of the ANN model to predict pansteatitis using blood chemistry parameters. The ROC curve in Fig 3A indicates that the blood chemistry data can correctly classify pansteatitis-affected tilapia with an accuracy of 85% using the blood chemistry values (with age and total length included). Further, the figure also suggests that false positives and false negative are unlikely, with false positives somewhat more likely than false negatives. A ROC curve using only the principle drivers for correct non-lethal (excluding age) classification of pansteatitis (Ca^2+^, Na^+^, TP and ALB with total length) was also constructed (Fig 3B) and resulted in providing a predictive power of 92%.

**Fig 4:**
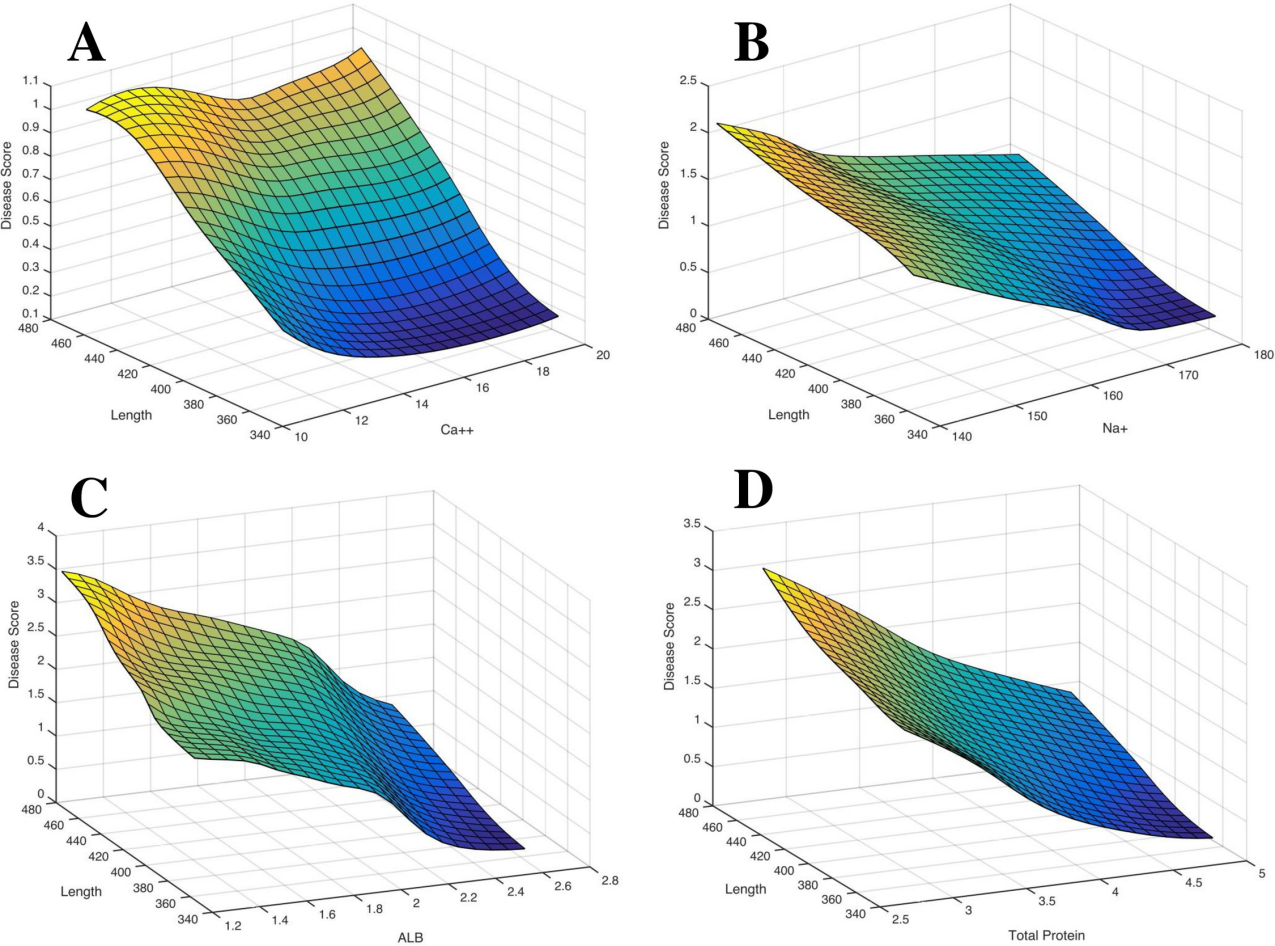
Surface plots for the four predictive parameters. A) Ca^2+^ (mg/dL), B) Na^+^ (mmol/L), C) ALB (g/dL), and D) TP (g/dL), in relation to vet score and total length (while keeping the other three parameters clamped).

Surface plots were created to help reveal more information about the most predictive blood chemistry parameters (Ca^2+^, Na^+^, ALB, TP with total length), as determined from the sensitivity plot shown in Fig 2. As shown in Fig 4A, as tilapia become longer in length (cm), there is a higher incidence of disease regardless of the Ca^2+^ levels, while Na^+^ (Fig 4B) shows an opposite effect in that the tilapia are more likely to be diseased at low Na^+^ levels (despite the total length). For ALB (Fig 4C) and TP (Fig 4D), tilapia are shown to have a high disposition of disease at lower blood chemistry values with the likelihood of having pansteatitis increasing with total length. In an effort to evaluate the overall distribution of the tilapia sample set, surface plots were created examining the four predictive parameters (Ca^2+^, Na^+^, ALB, and TP) in relation to length and standard deviation, as shown in S1 Fig. As shown in the figure, the plots indicate that to improve the predictive capabilities of using the ANN model, additional fish are required at all length classes (with a slightly greater need for larger fish).

## Discussion

Though recent reports have indicated that the disease could be increasing within the region [6], limited information is available regarding the exact presence of pansteatitis in fish populations within the Olifants River region. For any effective management plan of environmental disease to occur, the disease must first be well characterized within the region. Few efforts have been made to characterize the presence of pansteatitis in the region, in large part due to a lack of non-lethal approaches capable of providing high-throughput classification of pansteatitis status on a population-wide scale. In pursuit of establishing a non-invasive diagnostic approach, blood chemistry was evaluated as an approach to ascertain health status of fish potentially affected by pansteatitis. In this study, employment of a commercially available blood chemistry analyzer was evaluated in its ability to accurately classify healthy and disease status in tilapia. Several parameters in the tilapia plasma had a significant difference when comparing healthy and pansteatitis-affected tilapia using standard t-tests. Specifically, GLOB (p < 0.0001), ALB (males p < 0.0001, females p = 0.0091), TP (χ^2^ < 0.0001), PHOS (males p < 0.0001), and Ca^2+^ (males χ^2^ = 0.0004) had the strongest significance, followed by Na^+^ (p = 0.0116). Total protein, ALB, and GLOB were all lower in the pansteatitis-affected tilapia in comparison to healthy counterparts. Total protein, ALB, and GLOB have been previously associated with poor nutrition and starvation in some fish species [39, 56]. Further, previous reports have noted that animals with pansteatitis incur pain and a decreased ability to move as a result of the condition [14, 21, 57]. This, combined with the fact that tilapia can survive long periods of time without feeding [58], suggests that the lack of mobility could have an impact on feeding and subsequently the resultant blood chemistry values. Beyond diet-related effects, decreased GLOB levels in plasma have been previously associated with acute exposure to certain toxic chemicals [59–61] and low values in the pansteatitis-affected fish could be indicative of liver or renal damage [62, 63]. Total protein and ALB in plasma and serum have been regarded as indicators of liver and/or renal health in vertebrates [64, 65], and in toxicological studies with Nile Tilapia (*Oreochromis niloticus*), have been shown to decrease with insecticide exposure [66]. Interestingly, Ca^2+^ in plasma only exhibited a significant difference in males of healthy and pansteatitis-affected tilapia (male χ^2^ = 0.0004, female: χ^2^ = 0.1016). Disturbance in Ca^2+^ regulation has been shown previously to lead to oxidative stress and toxicity [67–70]. In summary, the combined data suggests that pansteatitis-affected fish could have altered regulation of ion balance (as shown by the lowered circulating plasma Ca^2+^ (in males) and Na^+^ levels), altered metabolic response within the liver and renal systems (lower TP and PHOS), and altered dietary intake, in comparison to their healthy counterpart.

In addition to the blood chemistry, the morphometric traits of each fish were compared, and age, total length and weight were all shown to be significantly different when comparing healthy and pansteatitis-affected individuals. Previously, Huchzermeyer et al. found that histological signs of the disease were present in both sexes of sharptooth catfish, but were entirely absent in smaller individuals (< 2 kg total body mass and < 70 cm total body length) [12]. To date, few studies have associated age with pansteatitis. Most notably, a study by Huchzermeyer et al. demonstrated that age was not related with the disease in Sharptooth catfish from a large survey in KNP [6]. However, in our study at Lake Loskop, it was found that older (and longer and heavier) tilapia (average birth class of 2005) presented with pansteatitis more frequently than younger tilapia (average birth class of 2008). One hypothesis for this observation is that older tilapia could be more susceptible to pansteatitis (or triggers of pansteatitis) than younger tilapia. Two alternative hypotheses that could explain the higher incidence of pansteatitis with older tilapia is the concept of 1) a specific pansteatitis-causing event that potentially took place prior to the birth of the younger tilapia or 2) that with age comes an extended burden or increased lifetime of exposure to pansteatitis triggers.

While the primary goal of the study was to identify potential blood-based biomarkers of pansteatitis in tilapia, a secondary goal was to establish a model capable of using the blood chemistry results to non-lethally predict disease status. The ANN approach employed in this study was first introduced into clinical science in the mid 1990’s and has become something of a ‘gold standard’ for diagnosing disease using complex clinical data [71]. The algorithm has the advantage of being infinitely flexible, able to assume the shape of any unbroken curve, and able to incorporate a virtually unlimited number of diagnostic variables, making it ideal for evaluating the intricate relationship between blood chemistry parameters and each tilapia’s physical attributes and pansteatitis status. The high R-squared value for the constructed ANN model (0.93 ± 0.03) indicated that the disease designation of healthy (vet score <1) and pansteatitis-affected (vet score ≥ 1) was appropriate for proper disease classification. With age class and total length added to the ANN model, the predictive power was also assessed through cross validation (0.45 ± 0.08) and the resultant ROC curves. ROC curves are routinely used in clinical medicine to evaluate the effectiveness of diagnostic tests [72, 73]. A ROC curve provides a value that describes the accuracy (area under the curve) of a given model in separating two different groups (in this case, healthy and diseased) [74]. In assessing the accuracy of a diagnostic model, classification is typically defined by excellent (0.9–1), good (0.8–0.9), fair (0.7–0.8), poor (0.6–0.7) or fail (0.5–0.6) [75]. The ROC curve for the ANN model resulted in a predictive power of 85%, further supporting the assertion that the ANN model (with the blood chemistry data) described in this report holds considerable promise as a pansteatitis diagnostic tool.

After looking at the sensitivity plot (Fig 2), highlighting the most important parameters for non-invasively classifying disease, it was observed that four specific parameters (Ca^2+^, Na^+^, TP, ALB) and total length were the most influential (age was removed due to the lethality of otolith collection). Thus, a follow-up ANN model was constructed to determine the predictive power of only using the top four parameters with total length. The new ANN model resulted in a modest drop in the R-squared model value (0.8) and comparable R-squared cross-validation values (0.46). Interestingly, the area under the ROC curve increased to 0.92 ± 0.06 (92% predictive), leading to a modest increase in predictive capabilities (as shown in Fig 3B). This suggests that while all parameters could be used to predict pansteatitis, specifically for tilapia during this time period, it may be more efficient to only measure Ca^2+^, Na^+^, ALB and TP.

To examine the impacts of varying input variables on the expected disease status of the tilapia, artificial data sets were constructed that clamped all but two of the variables to their mean values and allowed the remaining variables to vary throughout their entire ranges in 5% increments. The variables chosen for this were total length, Ca^2+^, Na^+^, ALB, and TP, as these variables accounted for most of the explained variation in disease status (Fig 2). As shown in Fig 4A through 4D, smaller fish are expected to be generally healthier and have higher serum concentrations of Ca^2+^, Na^+^, ALB, and TP, than larger fish. Future studies will aim to sample a wider size (i.e., age) range to more thoroughly investigate this hypothesis and the potential presence of early stage markers of the disease.

One limitation of employing ANN projections as a means to predict disease status with artificial data is the assumption that the data used to generate the models has adequately sampled the variable space. To address this issue, a previously developed approach to assess adequacy of sampling was employed. As the ANN procedure was composed of 20 ANN runs with random sampling of the data records each run, it was reasoned that if multiple models generated consistent predictions for disease status, then the standard deviations among model predictions for some regions in the grid would approach zero and further sampling would be unnecessary in these regions [76]. Using the same artificial data employed to assess the predicted impacts of variables on disease status, the standard deviation of each of the points was plotted in the grids across all ANN models and the data is presented in S1 Fig. As can be seen, the standard deviation is low for small fish at higher serum concentrations of the variables. The standard deviation increases as serum concentrations decrease and in larger fish in general. As the size of the fish sampled can be controlled, this would suggest that sampling larger fish would provide more accurate models and improve the prediction of disease status.

## Conclusion

A current gap in the research of characterizing the environmental occurrence of pansteatitis is the lack of suitable approaches for surveying both the pervasiveness and prevalence of the disease over large-scale geographical regions or across species. In this study, blood chemistry was examined as a viable pansteatitis predictive matrix using a commercially available blood chemistry analyzer, which was capable of simultaneously measuring up to 12 unique biological parameters that have been previously associated with health and disease. Through initial testing, the blood chemistry device was found to be simple (automated), rapid (each test < 15 min), relatively inexpensive (≈ $12/rotor), efficient (12 parameters measured from 150 μL of plasma), compatible with tilapia plasma, reproducible (overall RSD of 4%) and relatively accurate (when compared to the noted values for SRM 1950); all characteristics that make the blood chemistry analyzer a suitable device for universal application of detecting environmental disease. Using t-tests, it was shown that the blood chemistry of diseased male tilapia were much more affected in comparison to the female tilapia, showing statistically significant differences in six blood chemistry parameters (in comparison to three in females). During t-test examination, it was also noted that all physical attributes (age, total length, and weight) were significantly different between healthy and pansteatitis-affected tilapia. Further, using a constructed ANN model aimed to classify disease status, several blood parameters (Ca^2+^, Na^+^, ALB and TP) along with total length were noted to be important variables in correctly classifying pansteatitis, with a predictive power of 92%, suggesting blood chemistry analysis using a blood chemistry analyzer holds considerable promise as a tool for routine population-wide pansteatitis assessments. Future studies are warranted to evaluate pansteatitis-related blood chemistry values beyond this study, specifically across different sites, seasons, and species.

## Supporting Information

S1 Fig Surface plots for the top four predictive parameters (Ca^2+^, Na^+^, ALB, and TP) in relation to standard deviation and total length (with the other three parameters were clamped). TP (g/dL), ALB (g/dL), Na^+^ (mmol/L), and Ca^2+^ (mg/dL).
(DOCX)

S1 Table 2014 Mozambique tilapia (*Oreochromis mossambicus*) morphometric information. (DOCX)

S2 Table Summarized male tilapia blood chemistry parameters using the blood chemistry analyzer. (DOCX)

S3 Table Summarized female tilapia blood chemistry parameters using the blood chemistry analyzer. (DOCX)

S4 Table Summarized trial examination of blood chemistry analysis using species-matched (tilapia) QC sample (n = 7). (DOCX)

S5 Table Summarized examination of SRM 1950 using a blood chemistry analyzer (n = 7). (DOCX)

S6 Table Blood chemistry values for SRM 1950 and comparison to noted NIST concentrations. (DOCX)
