# Supplementary material for: Predictive Blood Chemistry Parameters for Pansteatitis-Affected Mozambique Tilapia (Oreochromis mossambicus)
Source: PLoS One. 2016 Apr 26;11(4):e0153874. doi: 10.1371/journal.pone.0153874 (PMC4846142; doi:10.1371/journal.pone.0153874)
Supplement: S1 Fig — TP (g/dL), ALB (g/dL), Na+ (mmol/L), and Ca2+ (mg/dL). (DOCX) [file pone.0153874.s001.docx]

Supplemental Information for manuscript titled:

**Predictive Blood Chemistry Parameters for Pansteatitis-Affected Mozambique Tilapia (*Oreochromis mossambicus*)**

***John A. Bowden, Theresa M. Cantu, Robert W. Chapman, Stephen E. Somerville, Matthew P. Guillette, Hannes Botha, Andre Hoffman, Wilmien J. Luus-Powell, Willem J. Smit, Jeffrey Lebepe, Jan Myburgh, Danny Govender, Jonathan Tucker, Ashley S. P. Boggs, and Louis J. Guillette, Jr.**

*author to whom correspondence should be addressed


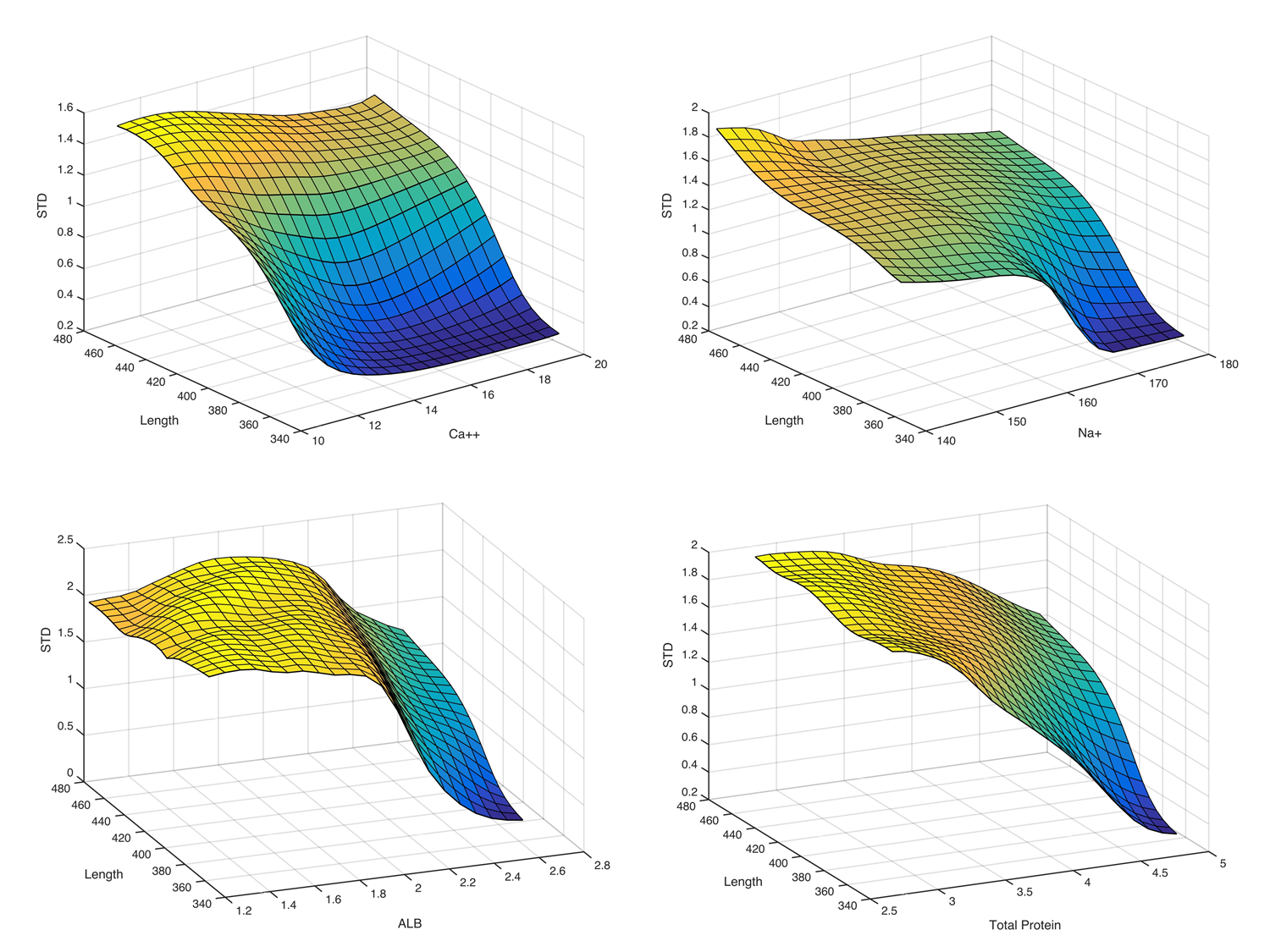


S1 Fig. Surface plots for the top four predictive parameters (Ca^2+^, Na^+^, ALB, and TP) in relation to standard deviation and total length (with the other three parameters were clamped). TP (g/dL), ALB (g/dL), Na^+^ (mmol/L), and Ca^2+^ (mg/dL).
