# Supplementary material for: Predictive Blood Chemistry Parameters for Pansteatitis-Affected Mozambique Tilapia (Oreochromis mossambicus)
Source: PLoS One. 2016 Apr 26;11(4):e0153874. doi: 10.1371/journal.pone.0153874 (PMC4846142; doi:10.1371/journal.pone.0153874)
Supplement: S1 Table — (DOCX) [file pone.0153874.s002.docx]

Supplemental Information for manuscript titled:

**Predictive Blood Chemistry Parameters for Pansteatitis-Affected Mozambique Tilapia (*Oreochromis mossambicus*)**

***John A. Bowden, Theresa M. Cantu, Robert W. Chapman, Stephen E. Somerville, Matthew P. Guillette, Hannes Botha, Andre Hoffman, Wilmien J. Luus-Powell, Willem J. Smit, Jeffrey Lebepe, Jan Myburgh, Danny Govender, Jonathan Tucker, Ashley S. P. Boggs, and Louis J. Guillette, Jr.**

*author to whom correspondence should be addressed

S1 Table. 2014 Mozambique tilapia (*Oreochromis mossambicus*) morphometric information

| **Fish** | **Date** |  | **Weight** | **Length** | **Vet Score** | **Age** |
| --- | --- | --- | --- | --- | --- | --- |
| **ID** | **Captured** | **Sex** | **(kg)** | **(cm)** | **(0-5)** | **(years)** |
| 8652 | 7/28/2014 | M | 2.10 | 44.7 | 0 | 5 |
| 8656 | 7/28/2014 | F | 1.60 | 43.0 | 2 | 10 |
| 8657 | 7/28/2014 | F | 1.65 | 41.0 | 2 | N/A |
| 8658 | 7/29/2014 | M | 2.35 | 46.0 | 5 | 8 |
| 8659 | 7/29/2014 | M | 1.20 | 37.5 | 0 | 3 |
| 8660 | 7/29/2014 | M | 1.80 | 43.7 | 2 | 6 |
| 8662 | 7/29/2014 | F | 1.35 | 40.2 | 2 | 9 |
| 8663 | 7/29/2014 | M | 1.75 | 42.8 | 1 | 7 |
| 8664 | 7/29/2014 | M | 1.95 | 43.1 | 0.5 | 6 |
| 8665 | 7/29/2014 | M | 1.45 | 40.3 | 0.5 | 6 |
| 8666 | 7/29/2014 | M | 2.45 | 47.4 | 2 | 5 |
| 8668 | 7/29/2014 | M | 1.65 | 44.0 | 3 | 8 |
| 8669 | 7/29/2014 | F | 1.50 | 39.2 | 0.5 | 7 |
| 8670 | 7/29/2014 | M | 2.00 | 44.0 | 3 | 7 |
| 8671 | 7/29/2014 | F | 1.55 | 39.8 | 0.5 | 8 |
| 8672 | 7/29/2014 | F | 1.50 | 39.6 | 4 | 10 |
| 8676 | 7/30/2014 | M | 1.70 | 42.5 | 1 | 7 |
| 8677 | 7/30/2014 | F | 1.65 | 41.5 | 1 | 10 |
| 8678 | 7/30/2014 | M | 1.90 | 43.2 | 4.75 | 10 |
| 8679 | 7/30/2014 | M | 2.60 | 47.5 | 5 | 11 |
| 8680 | 7/30/2014 | F | 1.45 | 41.1 | 1.5 | 9 |
| 8681 | 7/30/2014 | M | 1.50 | 41.0 | 0 | 6 |
| 8682 | 7/30/2014 | F | 1.50 | 39.2 | 2 | 10 |
| 8683 | 7/30/2014 | M | 1.40 | 41.0 | 0 | 6 |
| 8684 | 7/30/2014 | F | 1.90 | 44.3 | 1.5 | 11 |
| 8686 | 7/30/2014 | F | 1.30 | 38.3 | 2 | 9 |
| 8690 | 7/31/2014 | M | 1.95 | 43.5 | 5 | 6 |
| 8691 | 7/31/2014 | F | 1.40 | 39.2 | 4 | 11 |
| 8692 | 7/31/2014 | M | 1.75 | 42.2 | 0.5 | 5 |
| 8693 | 7/31/2014 | F | 1.25 | 38.5 | 0.5 | 6 |
| 8694 | 7/31/2014 | F | 1.30 | 38.2 | 0 | 6 |
| 8695 | 7/31/2014 | F | 1.10 | 36.5 | 0 | 5 |
| 8696 | 7/31/2014 | F | 1.65 | 40.0 | 0.5 | 9 |

Vet scores were given on a scale of 0 – 5 (0 is completely healthy). N/A indicates that the otolith for this animal was not collected.

S1 Table. Continued.

| **Fish** | **Date** |  | **Weight** | **Length** | **Vet Score** | **Age** |
| --- | --- | --- | --- | --- | --- | --- |
| **ID** | **Captured** | **Sex** | **(kg)** | **(cm)** | **(0-5)** | **(years)** |
| 8697 | 7/31/2014 | F | 1.35 | 38.6 | 0.5 | 7 |
| 8698 | 7/31/2014 | F | 1.05 | 36.9 | 0.5 | 5 |
| 8699 | 7/31/2014 | M | 1.30 | 35.6 | 0.5 | 4 |
| 8700 | 7/31/2014 | F | 1.35 | 38.7 | 0.5 | 5 |
| 8701 | 7/31/2014 | F | 1.50 | 39.6 | 3.5 | 7 |
| 8709 | 7/31/2014 | M | 1.10 | 38.0 | 0 | 4 |

Vet scores were given on a scale of 0 – 5 (0 is completely healthy)
