# Supplementary material for: Predictive Blood Chemistry Parameters for Pansteatitis-Affected Mozambique Tilapia (Oreochromis mossambicus)
Source: PLoS One. 2016 Apr 26;11(4):e0153874. doi: 10.1371/journal.pone.0153874 (PMC4846142; doi:10.1371/journal.pone.0153874)
Supplement: S2 Table — (DOCX) [file pone.0153874.s003.docx]

Supplemental Information for manuscript titled:

**Predictive Blood Chemistry Parameters for Pansteatitis-Affected Mozambique Tilapia (*Oreochromis mossambicus*)**

***John A. Bowden, Theresa M. Cantu, Robert W. Chapman, Stephen E. Somerville, Matthew P. Guillette, Hannes Botha, Andre Hoffman, Wilmien J. Luus-Powell, Willem J. Smit, Jeffrey Lebepe, Jan Myburgh, Danny Govender, Jonathan Tucker, Ashley S. P. Boggs, and Louis J. Guillette, Jr.**

*author to whom correspondence should be addressed

S2 Table. Summarized male tilapia blood chemistry parameters using the blood chemistry analyzer

| **ID** | **Score** | **AST** | **BA** | **CK** | **GLU** | **Ca^2+^** | **PHOS** | **TP** | **ALB** | **GLOB** | **K^+^** | **Na^+^** |
| --- | --- | --- | --- | --- | --- | --- | --- | --- | --- | --- | --- | --- |
| 8683 | 0 | 198 | 0 | **N/A** | 32 | 14.1 | 9.1 | 3.7 | 2.0 | 1.7 | 5.4 | 167 |
| 8652 | 0 | 36 | 11 | 425 | 32 | 13.6 | 6.1 | 3.4 | 1.9 | 1.5 | 3.3 | 170 |
| 8681 | 0 | 39 | 0 | 1049 | 28 | 13.4 | 6.6 | 3.6 | 2.1 | 1.5 | 3.3 | 164 |
| 8709 | 0 | 60 | 4 | 670 | 31 | 15.0 | 8.1 | 3.9 | 2.2 | 1.7 | 4.8 | 179 |
| 8659 | 0 | 25 | 0 | 454 | 39 | 14.2 | 7.2 | 3.7 | 1.9 | 1.8 | 4.1 | 178 |
| 8665 | 0.5 | 41 | 0 | 423 | 31 | 14.9 | 8.2 | 4.2 | 2.2 | 2.0 | 4.4 | 167 |
| 8699 | 0.5 | 45 | 0 | 764 | 40 | 15.5 | 8.6 | 4.1 | 2.2 | 2.0 | 4.3 | 179 |
| 8692 | 0.5 | 187 | 0 | 4624 | 88 | 14.8 | 9.6 | 4.2 | 2.4 | 1.9 | 4.8 | 172 |
| 8664 | 0.5 | 46 | 0 | 2917 | 63 | 15.0 | 8.7 | 4.2 | 2.3 | 1.9 | 4.0 | 170 |
| 8663 | 1 | 26 | 0 | 531 | 32 | 13.7 | 6.2 | 3.1 | 1.7 | 1.4 | 3.5 | 171 |
| 8676 | 1 | 22 | 14 | 684 | 36 | 11.8 | 4.8 | 3.1 | 1.7 | 1.4 | 3.7 | 162 |
| 8666 | 2 | 36 | 0 | 1688 | 26 | 11.6 | 5.4 | 3.0 | 1.6 | 1.4 | 4.1 | 149 |
| 8660 | 2 | 49 | 0 | 2529 | 23 | 10.9 | 4.6 | 3.1 | 2.0 | 1.1 | 3.4 | 140 |
| 8670 | 3 | 95 | 0 | 3712 | 41 | 12.9 | 6.5 | 3.4 | 1.8 | 1.6 | 3.8 | 168 |
| 8668 | 3 | 59 | 94 | 3284 | 34 | 13.2 | 6.1 | 2.9 | 1.5 | 1.4 | 5.2 | 170 |
| 8678 | 4.75 | 21 | 0 | 598 | 35 | 12.4 | 4.5 | 3.3 | 1.6 | 1.7 | 3.8 | 164 |
| 8658 | 5 | 87 | 0 | 2886 | 27 | 11.6 | 4.1 | 2.9 | 1.5 | 1.4 | 4.7 | 160 |
| 8679 | 5 | 35 | 15 | 978 | 33 | 11.8 | 3.9 | 3.0 | 1.5 | 1.5 | 4.0 | 162 |
| 8690 | 5 | 206 | 14 | 4338 | 30 | 12.4 | 4.7 | 2.8 | 1.2 | 1.6 | 4.3 | 169 |

Uric acid measurement has been removed (not detected with tilapia). AST (U/L), TP (g/dL), ALB (g/dL), GLOB (g/dL), GLU (mg/dL), PHOS (mg/dL), K^+^ (mmol/L), Na^+^ (mmol/L), Ca^2+^ (mg/dL), BA (μmol/L), CK (U/L), and UA (mg/dL). U is equal to 16.67 nanokatals. N/A indicates that the VetScan analyzer did not provide a value.
