# Supplementary material for: Predictive Blood Chemistry Parameters for Pansteatitis-Affected Mozambique Tilapia (Oreochromis mossambicus)
Source: PLoS One. 2016 Apr 26;11(4):e0153874. doi: 10.1371/journal.pone.0153874 (PMC4846142; doi:10.1371/journal.pone.0153874)
Supplement: S3 Table — (DOCX) [file pone.0153874.s004.docx]

Supplemental Information for manuscript titled:

**Predictive Blood Chemistry Parameters for Pansteatitis-Affected Mozambique Tilapia (*Oreochromis mossambicus*)**

***John A. Bowden, Theresa M. Cantu, Robert W. Chapman, Stephen E. Somerville, Matthew P. Guillette, Hannes Botha, Andre Hoffman, Wilmien J. Luus-Powell, Willem J. Smit, Jeffrey Lebepe, Jan Myburgh, Danny Govender, Jonathan Tucker, Ashley S. P. Boggs, and Louis J. Guillette, Jr.**

*author to whom correspondence should be addressed

S3 Table. Summarized female tilapia blood chemistry parameters using the blood chemistry analyzer

| **ID** | **Score** | **AST** | **BA** | **CK** | **GLU** | **Ca^2+^** | **PHOS** | **TP** | **ALB** | **GLOB** | **K^+^** | **Na^+^** |
| --- | --- | --- | --- | --- | --- | --- | --- | --- | --- | --- | --- | --- |
| 8694 | 0 | 80 | 27 | 1742 | 33 | 18.4 | 8.3 | 4.9 | 2.4 | 2.5 | 5.3 | 170 |
| 8695 | 0 | 482 | 32 | 6925 | 47 | 18.9 | 11.1 | 4.6 | 2.6 | 1.9 | 5.6 | 176 |
| 8698 | 0.5 | 113 | 0 | 4039 | 27 | 14.8 | 7.7 | 3.9 | 2.3 | 1.6 | 4.5 | 167 |
| 8697 | 0.5 | 40 | 19 | 690 | 37 | 14.2 | 6.7 | 3.5 | 2.1 | 1.4 | 4.1 | 171 |
| 8669 | 0.5 | 34 | 0 | 746 | 27 | 14.9 | 7.4 | 3.7 | 2.1 | 1.6 | 3.1 | 177 |
| 8693 | 0.5 | 110 | 34 | 1308 | 30 | 15.2 | 7.7 | 4.0 | 2.3 | 1.7 | 5.0 | 168 |
| 8696 | 0.5 | 64 | 1 | 1669 | 27 | 15.3 | 5.1 | 3.2 | 1.9 | 1.3 | 3.8 | 155 |
| 8700 | 0.5 | 57 | 2 | 1922 | 38 | 16.0 | 7.5 | 4.2 | 2.5 | 1.8 | 4.0 | 170 |
| 8671 | 0.5 | 53 | 0 | 783 | 39 | 15.0 | 7.2 | 4.0 | 2.0 | 2.1 | 3.9 | 172 |
| 8677 | 1 | 31 | 0 | 1188 | 32 | 11.6 | 4.5 | 3.0 | 1.8 | 1.2 | 4.0 | 152 |
| 8684 | 1.5 | 175 | 10 | 4230 | 46 | 16.2 | 8.1 | 3.6 | 2.2 | 1.4 | 4.6 | 179 |
| 8680 | 1.5 | 21 | 0 | 683 | 29 | 14.5 | 5.5 | 2.9 | 1.9 | 1.0 | 4.0 | 165 |
| 8656 | 2 | 25 | 18 | 484 | 43 | 12.9 | 6.5 | 3.0 | 1.6 | 1.4 | 3.5 | 166 |
| 8662 | 2 | 47 | 0 | 2178 | 28 | 13.4 | 7.1 | 3.1 | 1.9 | 1.2 | 4.4 | 157 |
| 8682 | 2 | 45 | 0 | 3426 | 23 | 14.8 | 4.4 | 3.2 | 1.9 | 1.3 | 3.6 | 160 |
| 8657 | 2 | 55 | 46 | 2038 | 58 | 20.0 | 8.0 | 4.1 | 2.4 | 1.7 | 4.4 | 180 |
| 8686 | 2 | 33 | 0 | 1777 | 23 | 15.0 | 7.7 | 3.4 | 2.0 | 1.4 | 3.9 | 170 |
| 8701 | 3.5 | 92 | 18 | 2388 | 36 | 15.2 | 6.6 | 3.4 | 2.0 | 1.5 | 3.8 | 174 |
| 8691 | 4 | 117 | 4 | 4190 | 53 | 14.0 | 6.8 | 3.1 | 1.8 | 1.3 | 4.6 | 161 |
| 8672 | 4 | 31 | 0 | 284 | 23 | 14.8 | 5.2 | 3.1 | 1.9 | 1.3 | 4.1 | 165 |

Uric acid measurement has been removed (not detected with tilapia). AST (U/L), TP (g/dL), ALB (g/dL), GLOB (g/dL), GLU (mg/dL), PHOS (mg/dL), K^+^ (mmol/L), Na^+^ (mmol/L), Ca^2+^ (mg/dL), BA (μmol/L), CK (U/L), UA (mg/dL). U is equal to 16.67 nanokatals.
