# Supplementary material for: Predictive Blood Chemistry Parameters for Pansteatitis-Affected Mozambique Tilapia (Oreochromis mossambicus)
Source: PLoS One. 2016 Apr 26;11(4):e0153874. doi: 10.1371/journal.pone.0153874 (PMC4846142; doi:10.1371/journal.pone.0153874)
Supplement: S4 Table — (DOCX) [file pone.0153874.s005.docx]

Supplemental Information for manuscript titled:

**Predictive Blood Chemistry Parameters for Pansteatitis-Affected Mozambique Tilapia (*Oreochromis mossambicus*)**

***John A. Bowden, Theresa M. Cantu, Robert W. Chapman, Stephen E. Somerville, Matthew P. Guillette, Hannes Botha, Andre Hoffman, Wilmien J. Luus-Powell, Willem J. Smit, Jeffrey Lebepe, Jan Myburgh, Danny Govender, Jonathan Tucker, Ashley S. P. Boggs, and Louis J. Guillette, Jr.**

*author to whom correspondence should be addressed

S4 Table. Summarized trial examination of blood chemistry analysis using species-matched (tilapia) QC sample (n = 7)

| **ID #** | **AST** | **BA** | **CK** | **GLU** | **Ca^2+^** | **PHOS** | **TP** | **ALB** | **GLOB** | **K^+^** | **Na^+^** |
| --- | --- | --- | --- | --- | --- | --- | --- | --- | --- | --- | --- |
| 1111 | 93 | 5 | 1788 | 35 | 14.1 | 7.1 | 3.5 | 2 | 1.5 | 3.6 | 163 |
| 1112 | 95 | 6 | 1652 | 35 | 14.5 | 7.2 | 3.5 | 2 | 1.5 | 3.6 | 162 |
| 1113 | 95 | 6 | 1585 | 34 | 14.4 | 6.9 | 3.5 | 2 | 1.5 | 3.5 | 161 |
| 1114 | 92 | 5 | 1577 | 34 | 13.4 | 6.8 | 3.3 | 1.9 | 1.4 | 3.3 | 158 |
| 1115 | 94 | 6 | 1747 | 35 | 14.4 | 6.8 | 3.5 | 2 | 1.5 | 3.4 | 161 |
| 1116 | 94 | 6 | 1571 | 35 | 14.4 | 7.2 | 3.5 | 2 | 1.5 | 3.5 | 163 |
| 1117 | 95 | 6 | 1550 | 33 | 14.3 | 6.7 | 3.4 | 1.9 | 1.5 | 3.3 | 159 |
| Average | 94.0 | 5.7 | 1638.6 | 34.4 | 14.2 | 7.0 | 3.5 | 2.0 | 1.5 | 3.5 | 161.0 |
| St. Dev | 1.2 | 0.5 | 94.3 | 0.8 | 0.4 | 0.2 | 0.1 | 0.0 | 0.0 | 0.1 | 1.9 |
| RSD | 1% | 9% | 6% | 2% | 3% | 3% | 2% | 2% | 3% | 4% | 1% |

Uric acid measurement has been removed (not detected with tilapia QC pool). AST (U/L), TP (g/dL), ALB (g/dL), GLOB (g/dL), GLU (mg/dL), PHOS (mg/dL), K^+^ (mmol/L), Na^+^ (mmol/L), Ca^2+^ (mg/dL), BA (μmol/L), CK (U/L), UA (mg/dL). U is equal to 16.67 nanokatals.
