# Supplementary material for: Predictive Blood Chemistry Parameters for Pansteatitis-Affected Mozambique Tilapia (Oreochromis mossambicus)
Source: PLoS One. 2016 Apr 26;11(4):e0153874. doi: 10.1371/journal.pone.0153874 (PMC4846142; doi:10.1371/journal.pone.0153874)
Supplement: S5 Table — (DOCX) [file pone.0153874.s006.docx]

Supplemental Information for manuscript titled:

**Predictive Blood Chemistry Parameters for Pansteatitis-Affected Mozambique Tilapia (*Oreochromis mossambicus*)**

***John A. Bowden, Theresa M. Cantu, Robert W. Chapman, Stephen E. Somerville, Matthew P. Guillette, Hannes Botha, Andre Hoffman, Wilmien J. Luus-Powell, Willem J. Smit, Jeffrey Lebepe, Jan Myburgh, Danny Govender, Jonathan Tucker, Ashley S. P. Boggs, and Louis J. Guillette, Jr.**

*author to whom correspondence should be addressed

S5 Table. Summarized examination of SRM 1950 using a blood chemistry analyzer (n = 7)

| **ID #** | **AST** | **CK** | **UA** | **GLU** | **Ca^2+^** | **PHOS** | **TP** | **ALB** | **GLOB** | **K^+^** | **Na^+^** |
| --- | --- | --- | --- | --- | --- | --- | --- | --- | --- | --- | --- |
| 2222 | 20 | 100 | 3.5 | 83 | 7.6 | 3.4 | 6.1 | 3.9 | 2.2 | 3.7 | 133 |
| 2223 | 20 | 93 | 3.4 | 83 | 7.7 | 3.5 | 6.1 | 3.9 | 2.2 | 3.8 | 139 |
| 2224 | 18 | 93 | 3.4 | 84 | 7.6 | 3.4 | 6.1 | 3.9 | 2.1 | 3.7 | 138 |
| 2225 | 19 | 96 | 3.1 | 80 | 7.3 | 3.1 | 5.8 | 3.8 | 2.1 | 3.4 | 132 |
| 2226 | 21 | 101 | 3.4 | 83 | 7.5 | 3.3 | 6 | 3.9 | 2.1 | 3.8 | 138 |
| 2227 | 22 | 111 | 3.5 | 83 | 7.7 | 3.3 | 6 | 3.9 | 2.2 | 3.8 | 140 |
| 2228 | 21 | 106 | 3.5 | 84 | 7.6 | 3.9 | 6.1 | 4 | 2.1 | 3.5 | 140 |
| Average | 20.1 | 100.0 | 3.4 | 82.9 | 7.6 | 3.4 | 6.0 | 3.9 | 2.1 | 3.7 | 137.1 |
| St Dev | 1.3 | 6.7 | 0.1 | 1.3 | 0.1 | 0.2 | 0.1 | 0.1 | 0.1 | 0.2 | 3.3 |
| RSD | 7% | 7% | 4% | 2% | 2% | 7% | 2% | 1% | 2% | 4% | 2% |

Bile acid measurement has been removed (not detected with SRM 1950). AST (U/L), TP (g/dL), ALB (g/dL), GLOB (g/dL), GLU (mg/dL), PHOS (mg/dL), K^+^ (mmol/L), Na^+^ (mmol/L), Ca^2+^ (mg/dL), BA (μmol/L), CK (U/L), UA (mg/dL). U is equal to 16.67 nanokatals.
