# Supplementary material for: Predictive Blood Chemistry Parameters for Pansteatitis-Affected Mozambique Tilapia (Oreochromis mossambicus)
Source: PLoS One. 2016 Apr 26;11(4):e0153874. doi: 10.1371/journal.pone.0153874 (PMC4846142; doi:10.1371/journal.pone.0153874)
Supplement: S6 Table — (DOCX) [file pone.0153874.s007.docx]

Supplemental Information for manuscript titled:

**Predictive Blood Chemistry Parameters for Pansteatitis-Affected Mozambique Tilapia (*Oreochromis mossambicus*)**

***John A. Bowden, Theresa M. Cantu, Robert W. Chapman, Stephen E. Somerville, Matthew P. Guillette, Hannes Botha, Andre Hoffman, Wilmien J. Luus-Powell, Willem J. Smit, Jeffrey Lebepe, Jan Myburgh, Danny Govender, Jonathan Tucker, Ashley S. P. Boggs, and Louis J. Guillette, Jr.**

*author to whom correspondence should be addressed

S6 Table. Blood chemistry values for SRM 1950 and comparison to noted NIST concentrations

| **SRM 1950** | **Vet Scan** | | | **Certified Value** | | |  |
| --- | --- | --- | --- | --- | --- | --- | --- |
| **Certified Data** |  |  |  |  |  |  | **95 % CI** |
| Glucose (mg/dL) | 82.86 | ± | 1.34 | 82.16 | ± | 1.00 | Within |
| Uric Acid (mg/dL) | 3.40 | ± | 0.14 | 4.274 | ± | 0.089 | Below |
| Calcium (mmol/L) | 1.89 | ± | 0.03 | 1.936 | ± | 0.024 | Within |
| Potassium (mmol/L) | 3.67 | ± | 0.16 | 3.665 | ± | 0.025 | Within |
| Sodium (mmol/L) | 137.14 | ± | 3.29 | 141.76 | ± | 0.31 | Below |
| Total Protein (g/L)* | 60.29 | ± | 1.11 | 59.1 | ± | 1.7 | Within |
|  |  |  |  |  |  |  |  |
| **SRM/D Clinical Data†** |  |  |  |  |  |  | **% Difference** |
| Aspartate Aminotransferase (U/L) | 20.14 | ± | 1.34 | 18.8 | ± | 0.4 | 6.8% |
| Creatine Kinase (U/L) | 100 | ± | 6.73 | 89.8 | ± | 2.5 | 10.7% |
| Albumin (g/dL) | 3.90 | ± | 0.06 | 3.6 | ± | 0.1 | 8.0% |
|  |  |  |  |  |  |  |  |
| **Calculated Data††** |  |  |  |  |  |  | **% Difference** |
| Globulin (g/dL) | 2.10 | ± | 0.1 | 2.31 | ± | 0.19 | 9.5 % |

* Indicates value noted is a reference value, † Indicates value noted is an averaged value obtained online from the SRM/D website. CI indicates the 95 % confidence interval of the reported NIST value. †† Indicates calculated NIST value was obtained by subtracting the albumin value from the total protein value. The blood chemistry device did not provide a bile acid measurement for SRM 1950. There is no certified SRM 1950 value noted for phosphorous.
